# Cell-Surface Receptors Transactivation Mediated by G Protein-Coupled Receptors

**DOI:** 10.3390/ijms151119700

**Published:** 2014-10-29

**Authors:** Fabio Cattaneo, Germano Guerra, Melania Parisi, Marta De Marinis, Domenico Tafuri, Mariapia Cinelli, Rosario Ammendola

**Affiliations:** 1 Department of Molecular Medicine and Medical Biotechnology, School of Medicine, University of Naples Federico II, Naples 80131, Italy; E-Mails: fabio.cattaneo@unina.it (F.C.); melania.parisi@unina.it (M.P.); mar.demarinis@studenti.unina.it (M.D.M.); 2 Department of Medicine and Health Sciences, University of Molise, Campobasso 86100, Italy; E-Mail: germano.guerra@unimol.it; 3 Department of Sport Science and Wellness, University of Naples Parthenope, Naples 80133, Italy; E-Mail: tafuri@uniparthenope.it; 4 Department of Public Health, School of Medicine, University of Naples Federico II, Naples 80131, Italy; E-Mail: mariapia.cinelli@unina.it

**Keywords:** GPCR, tyrosine kinase receptor, transactivation, cell signaling, reactive oxygen species

## Abstract

G protein-coupled receptors (GPCRs) are seven transmembrane-spanning proteins belonging to a large family of cell-surface receptors involved in many intracellular signaling cascades. Despite GPCRs lack intrinsic tyrosine kinase activity, tyrosine phosphorylation of a tyrosine kinase receptor (RTK) occurs in response to binding of specific agonists of several such receptors, triggering intracellular mitogenic cascades. This suggests that the notion that GPCRs are associated with the regulation of post-mitotic cell functions is no longer believable. Crosstalk between GPCR and RTK may occur by different molecular mechanism such as the activation of metalloproteases, which can induce the metalloprotease-dependent release of RTK ligands, or in a ligand-independent manner involving membrane associated non-receptor tyrosine kinases, such as c-Src. Reactive oxygen species (ROS) are also implicated as signaling intermediates in RTKs transactivation. Intracellular concentration of ROS increases transiently in cells stimulated with GPCR agonists and their deliberated and regulated generation is mainly catalyzed by enzymes that belong to nicotinamide adenine dinucleotide phosphate (NADPH) oxidase family. Oxidation and/or reduction of cysteine sulfhydryl groups of phosphatases tightly controls the activity of RTKs and ROS-mediated inhibition of cellular phosphatases results in an equilibrium shift from the non-phosphorylated to the phosphorylated state of RTKs. Many GPCR agonists activate phospholipase C, which catalyze the hydrolysis of phosphatidylinositol 4,5-bis-phosphate to produce inositol 1,4,5-triphosphate and diacylglicerol. The consequent mobilization of Ca^2+^ from endoplasmic reticulum leads to the activation of protein kinase C (PKC) isoforms. PKCα mediates feedback inhibition of RTK transactivation during GPCR stimulation. Recent data have expanded the coverage of transactivation to include Serine/Threonine kinase receptors and Toll-like receptors. Herein, we discuss the main mechanisms of GPCR-mediated cell-surface receptors transactivation and the pathways involved in intracellular responses induced by GPCR agonists. These studies may suggest the design of novel strategies for therapeutic interventions.

## 1. Introduction

Cross-communication between different signaling systems plays a key role to coordinate the plethora of extracellular stimuli to which a cell is subjected under several physiological or pathological conditions. Cell-surface receptors are the key components of these networks and the inter-receptor crosstalk acts as a general signaling mechanism connecting and diversifying signal transduction pathways. The major classes of cell surface transmembrane proteins are tyrosine kinase receptors (RTKs) and G-protein-coupled receptors (GPCRs), which are the largest group of cell-surface seven-transmembrane proteins [1]. RTKs activation is achieved by ligand binding to the extracellular domain, which can induce dimerization of the receptor and, in turn, the autophosphorylation on tyrosine residues within the cytosolic domain with the formation of Src homology 2 (SH2) or phospho-tyrosine binding (PTB) sites [2]. These represent docking sites for the recruitment of SH2-domain-containing proteins or adaptor proteins, which trigger intracellular signaling cascades. Some signaling proteins containing SH2-domains possess intrinsic tyrosine kinase activity (Src kinases), other adaptor proteins utilize their SH2 and SH3 domains to mediate interactions with protein involved in signal transduction, as the case of growth factor receptor-bound protein 2 (Grb2) which recruits Son of sevenless (Sos) protein and triggers the Ras pathway, which leads to phosphorylation and activation of the serine–threonine kinase MAPK (Mitogen-activated protein kinase) [2]. Moreover, binding of hepatocyte growth factor (HGF) to c-Met, a member of the RTK family, induces serine phosphorylation of Smads signaling proteins [3]. GPCRs lack intrinsic enzymatic activity and are coupled to heterotrimeric G proteins, which consist of Gα, Gβ and Gγ subunits. Ligand binding stabilizes the occupied GPCR in an active signaling conformation during which the heterotrimeric G-proteins dissociate in GTP-bound Gα and Gβγ subunits. These regulate the activity of several enzymes such as adenylate cyclase, phospholipase C (PLC) isoforms and kinases, resulting in generation of intracellular second messengers that control cellular functions. Adding to the complexity of G protein-dependent signaling is the existence of four major members of the Gα subunit family: Gα_s_, Gα_i_, Gα_12/13_ and Gα_q_, which are responsible for triggering different signaling responses. Currently 20 Gα, 6 Gβ and 11 Gγ subunits have been identified [4].

The activity of most GPCRs are regulated by GPCR kinases (GRKs) that phosphorylate the *C*-terminal tail of activated GPCRs, preventing further interaction with heterotrimeric G proteins and leading to termination of receptor signaling and receptor desensitization [5]. GPCR phosphorylation also facilitate recruitment of arrestin proteins (β-arrestin), which bind to the desensitized receptors allowing endocytosis via clathrin-coated pits [6]. During desensitization and endocytosis, arrestins also act as scaffolding proteins to recruit a series of signaling and regulatory proteins that add spatiotemporal complexity to GPCR functions.

RTKs and GPCRs act in concert to regulate physiological processes and in some cases their effects are synergic whereas in others they antagonize [7]. The activation of GPCRs can stimulate the signaling activity of RTKs connecting the broad diversity of GPCRs with the potent signaling capacities of RTKs. This molecular mechanism is termed transactivation and was first described in Rat-1 fibroblasts stimulated with a number of GPCR agonists, which induced a rapid tyrosine phosphorylation of epidermal growth factor receptor (EGFR) [8]. GPCR-induced EGFR tyrosine phosphorylation is rapid, transient, and comparable with receptor activation by low amounts of EGF [9].

Moreover, recent evidence show that RTKs use a G protein to induce activation of signaling pathways [10], and that several GPCR agonists can transactivate members of the cell surface transforming growth factor (TGF)-β receptors superfamily, which possess predominantly Serine/Threonine kinase (S/TK) activity [11], as well as Toll-like receptors (TLRs).

Transactivation of RTKs by GPCRs signaling can occur through different mechanisms. In the ligand-dependent triple-membrane-passing-signal (TMPS) mechanism, GPCR-mediated RTK transactivation depends on activation of membrane-bound matrix metalloproteases (MMPs), such as the A Disintegrin And Metalloprotease (ADAM) family members [9,12]. The ligand-independent mechanism suggests that GPCR stimulation triggers the activation of several second messengers such as Ca^2+^ ions, protein kinase C (PKC), the non-receptor protein tyrosine kinases Src and Pyk, β-arrestin and reactive oxygen species (ROS) which, in turn, induce tyrosine phosphorylation and subsequent activation of RTKs [13,14,15,16,17,18]. Herein, we present a general overview of the diverse mechanisms contributing to the crosstalk between GPCRs and RTKs.

## 2. Ligand-Dependent Triple-Membrane-Passing-Signal (TMPS) Mechanisms: Role of Membrane-Bound Matrix Metalloproteases (MMPs) and the A Disintegrin and Metalloproteases (ADAMs)

Matrix metalloproteases belong to the calcium-dependent endopeptidases family. The members of this family are structurally and functionally related and are secreted in an inactive form (pro-MMPs), which requires an activation step before they are able to cleave extracellular matrix (ECM) components. Other members of metalloproteases include ADAMs and ADAMs with Thrombospondin motifs (ADAMTs) enzymes. The cleavage of ECM by MMPs triggers cell migration [19] and the liberation of proforms of cytokines, growth factors and chemokines anchored to the plasma membrane [20,21].

**Figure 1:**
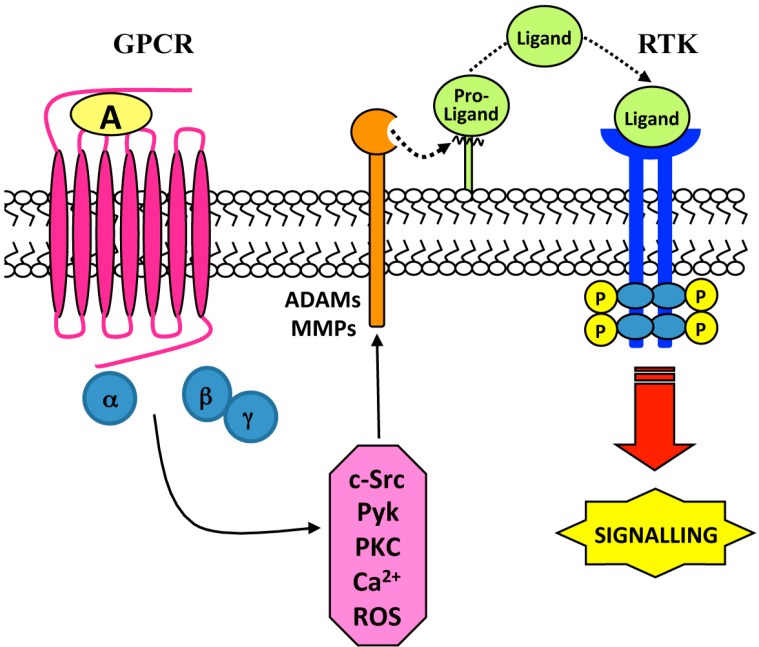
Ligand-dependent tyrosine kinase receptor (RTK) transactivation. Stimulation of G-protein-coupled receptors (GPCRs) with a selective agonist (A) triggers different intracellular signaling mediators via activation of Gα and/or Gβγ subunits. Metalloprotease-mediated proteolytic cleavage of a Pro-Ligand generates a Ligand which binds and transactivates RTK.

Several MMPs are involved in GPCR-mediated transactivation of different RTKs such as epidermal growth factor receptor (EGFR) [22,23,24,25], vascular endothelial growth factor receptor (VEGFR) [26,27,28] and platelet-derived growth factor receptor (PDGFR) [29,30], and this process is linked to MMP-mediated shedding of ligands, which in turn activate growth factor receptors (Figure 1). Several members of the MMP family are involved in the ectodomain shedding of EGFR ligands and, in turn, in EGFR transactivation. MMP-2 and MMP-9 represent important regulators of EGFR ligand release in isolated preovulatory ovarian follicles stimulated with the pituitary peptide—hormon luteinizant (LH) [31] and in gonadotropin-releasing hormone-stimulated (GnRH) gonadotropic cells [32], whereas MMP-7 mediates heparin-binding EGF-like growth factor (HB-EGF) shedding and EGFR transactivation in phenylephrine-stimulated arteries [33]. In some cases MMPs activation is downstream to EGFR transactivation. In fact, histamine stimulation of the histamine 2 (H2) receptor, a member of the GPCR family, evokes MMP-1 secretion that is dependent on the EGFR transactivation, as demonstrated by the inhibitor effect of the EGFR tyrosine kinase inhibitor AG1478 on the histamine-mediated MMP-1 secretion [34]. Moreover, in cultured chondrocytes thrombin increases the expression of MMP-13 by interacting with members of protease activated receptors (PARs) [35]. Another molecular signaling platform contemplates a neurominidase-1 (Neu-1)-MMP-9 crosstalk in alliance with the GPCR neuromedin B in activating EGFR. Central to this process is that Neu-1 and MMP-9 form a complex tethered at the ectodomain of EGFRs on the cell surface and that EGF binding induces a conformational change of EGFR to trigger MMP-9 activation and, in turn, Neu-1 stimulation. α-2,3-sialyl residues associated to β-galactosides, elsewhere from the EGF binding site, are hydrolysed by activated Neu1 [24]. In other examples the members of the MMP family involved in EGFR transactivation are not yet characterized. In fact, in human colonic myofibroblasts (18Co) cells chronic exposure to tumor necrosis factor (TNF-α) drives to up-regulation of EGFR in association with sustained lysophosphatidic acid (LPA)-mediated EGFR phosphorylation at Y1068, which is prevented by MMP inhibitors [36]. In cell culture models LPA also enhances corneal epithelial wound healing, which is prevented by tyrphostin, by MMPs inhibitors or by an HB-EGF antagonist. These inhibitors retard LPA-induced activation of EGFR and its downstream effectors extracellular-signal-regulated-kinases (ERKs) and Akt. The level of LPA-dependent EGFR phosphorylation is similar to that induced by wounding. However, LPA appears to prolong wound-induced EGFR signaling through its ability to induce autocrine HB-EGF signaling [37].

These data suggest the possible participation of MMPs in GPCR-induced EGFR transactivation in some settings and underline the ability of some MMPs to cleave proHB-EGF to produce mature HB-EGF (Table 1).

Agonist-bound GPCRs also activate members of the ADAM family, which in turn may transactivate different RTKs in several cell lines. ADAMs sheddase are a family of metalloproteases able to generate many diverse bioactive cytokines and growth factors by ectodomain shedding and to regulate many important cellular processes as growth, adhesion and motility of cells. Ectodomain cleavage is made specific by a number of intracellular signals, such as calcium influx, activation of GPCRs (Figure 1), and the release of diacylglycerol [38,39]. In the human genome 12 functional ADAMs have been identified and only ADAM10, ADAM12 and ADAM17 are the sheddases of the EGFR ligands in response to various shedding stimulants, such as GPCR agonists, growth factors, phorbol esters and cytokines [40,41]. The specificity and regulation of ADAMs involved in RTK transactivation is complex and depends on GPCR agonists and cell types under investigation. In cardiomiocytes Angiotensin II (Ang II) binds to Ang II type 1 (AT1) receptor, belonging to the GPCR family, and induces EGFR transactivation through HB-EGF shedding mediated by ADAM17 [42]. In kidney cancer cell lines, LPA-induced HB-EGF shedding and subsequent EGFR transactivation is mediated by ADAM10 in ACHN cells, whereas ADAM17 is responsible for these events in human renal carcinoma cells (CaKi2) and Human kidney carcinoma cell line (A498) [43]. In human tongue epithelial carcinoma cells (SCC-9) squamous carcinoma cell line ADAM17 mediates also amphiregulin shedding and EGFR transactivation by LPA [44] and in human urinary bludder epithelial cells (TccSup) ADAM15 mediates LPA-induced shedding of TGF-β [43]. In general ADAMs are required in EGFR transactivation by GPCRs and only few exceptions demonstrate the involvement of MMPs in this process.

GPCR stimulation results in ADAM-dependent shedding also of other proteins, such as interleukin-6 (IL-6) receptor shedding in neutrophils [45], TNF-α shedding through ADAM17 in neuronal cells [46], ADAM10/17-dependent shedding of amyloid precursor protein in astrocytoma cells [47]. ADAM10/17 stimulation generates also the chemokine family of the GPCR ligands (CX3CL1 (Chemokine (C-X3-C motif) ligand 1), CXCL-16 (Chemokine (C-X-C motif) ligand 16)) by shedding in several cellular systems [41]. The chemokine interleukin-8 (IL-8) binds to interleukin 8 receptor alpha (IL-8RA) (Chemokine receptor type 1, CXCR1) and Interleukin 8 receptor beta (IL-8RB) (Chemokine receptor type 2, CXCR2), which are members of the GPCR family. In colon cancer cells, stimulation of these receptors induces shedding of EGF ligands via activation of ADAMs with subsequent EGFR tyrosine phosphorylation [48]. In general, ADAM10 and -17 cleave most disease-relevant substrates and their general inhibitors do not have a significant clinical application. Since it is not possible to target and to cleave specific substrates, it is necessary to identify signaling intermediates that determine substrate specificity of cleavage [40].

**Table 1:** RTKs transactivation: role of metalloproteases.

Cell Lines	Stimulus	GPRC	Metalloproteases	RTK	Biological Responses	Ref.
Pancreatic cancer cells	EGF	Neuromedin B	MMP-9	EGFR	EGFR transactivation, cancer growth and metastatic spread	[24]
Isolated preovulatory ovarian follicles, Y1 adrenal cells	LH	LHRH	MMP-2–9	EGFR	EGFR transactivation, steroidogenesis	[31]
Gonadrotropic cells	GnRH	GnRHR	MMP-2–9	EGFR	EGFR transactivation, Src, Ras and ERKs activation	[32]
Mesenteric arteries	Phenylephrine	α1B-Adrenoreceptor	MMP-7	EGFR	EGFR transactivation, vasoconstriction, growth	[33]
Gastrics epithelial cells	Histamine	H2R	MMP-1	EGFR	EGFR transactivation, MAPK activation	[34]
Chondrocytes	Thrombin	PARs	MMP-13	EGFR	EGFR transactivation, PI3K/Akt pathway and AP1 activation	[35]
18Co	LPA, TNF-α	LPA1	MMP	EGFR	EGFR transactivation, MAPK phosphorylation, COX2 expression	[36]
Corneal epithelial cells	LPA	LPA1	MMP	EGFR	EGFR transactivation, ERK-Akt activation, wound healing, proliferation	[37]
Cardiomyocytes	Ang II	AT1	ADAM17	EGFR	EGFR transactivation, MAPK activation, angiogenesis	[42]
Kidney cancer cells, Bludder carcinoma cells, Caki2, A498, TccSup	LPA	LPA1	ADAM10–15–17	EGFR	EGFR transactivation, MAPK activation, tumor cell migration and invasion, TGF-β shedding	[43]
SCC-9	LPA, Carbachol	LPA, AChR	ADAM17	EGFR	EGFR transactivation, amphiregulin shedding, ERKs activation, PI3K/Akt activation, cell proliferation, migration	[44]
Neuroectodermal cells	Serotonin, Nor-epinephrine	5-HT2B, α1D-Adrenoreceptor	ADAM17	EGFR	EGFR transactivation, NADPH oxidase activation	[46]
Astrocytoma cells	UTP	P2Y2R	ADAM10–17	EGFR	EGFR transactivation, amyloid precursor shedding	[47]
Colon cancer cells	Interleukin-8	CXCR1, CXCR2	ADAMs	EGFR	EGFR transactivation, MAPK activation, cell growth	[48]
CHO, EC-4 ^(TACE+/+)^ EC-2 ^(TACE ΔZn/ΔZn)^	ATP	P2Y2R	ADAM17	EGFR	EGFR transactivation	[49]

18Co, Human colonic myofibroblasts; 5-HT2B, Serotonin receptor 2B; A498, Human kidney carcinoma cell line; AChR, Acetylcholine receptor; Ang II, Angiotensin II; AT1, Angiotensin II receptor type 1; ATP, Adenosine triphosphate; Caki2, Human renal carcinoma cells; CHO, Chinese hamster ovary cell line; CXCL12, Chemokine 12; CXCR1, Chemokine receptor type 1; CXCR2, Chemokine receptor type 2; CXCR4, Chemokine receptor type 4; EC-4 ^(TACE +/+)^ and EC-2 ^(TACE ΔZn/ΔZn)^, Mice fibroblasts cell lines; EGF, Epidermal growth factor; EGFR, Epidermal growth factor receptor; GnRH, Gonadotropin-releasing hormone; GnRHR, Gonadotropin-releasing hormone receptor; H2R, Histamine receptor; LH, Hormon luteinizant; LHRH, Luteinizing-hormone receptor; LPA, Lysophosphatidic acid; LPA1, Lysophosphatidic acid receptor type 1; N15C6, Human prostate epithelial cells; P2Y2R, Nucleotide receptor; PARs, Protease activated receptors; SCC-9, Human tongue epithelial carcinoma cells; TccSup, Human urinary bludder epithelial cells; TNF-α, Tumor necrosis factor; UTP, Uridine triphosphate.

Both MMPs and ADAMs have been implicated in ectodomain shedding of HB-EGF induced by ROS [50], which may oxidize the electrophilic thiol groups in the pro-domain of ADAMs and disrupt the cysteine–zinc bond, leading to the active form of the enzyme [9]. In fact, in tumor cells oxidative stress induces ADAM-9, -10, and -17 activity which results in cell-surface cleavage of pro-HB-EGF and subsequent EGFR transactivation [51] and adenosine triphosphate (ATP)-dependent activation of the purinergic receptor of the P2Y family of GPCRs stimulates TGFα proteolysis with concomitant EGFR activation, in both murine fibroblasts and Chinese hamster ovary (CHO) cells [49]. This process requires ADAM17 activity and ROS production by mitochondrial oxidative complex [49] (Table 1).

## 3. Ligand-Independent Mechanisms: Role of Reactive Oxygen Species (ROS)

Phosphorylation of receptor and non-receptor tyrosine kinases is a critical step in signal transduction that drives to specific cellular functions. ROS can activate many protein tyrosine kinases through different mechanisms [52]. In fact, ROS (1) may directly activate kinases by altering protein–protein interactions; (2) may directly inactivate by oxidation the cysteine residue in the catalytic site of protein tyrosine phosphatases, which in turn results in tyrosine kinases activation; (3) may stimulate proteolysis of regulatory proteins inhibiting tyrosine kinase activity [53].

**Figure 2:**
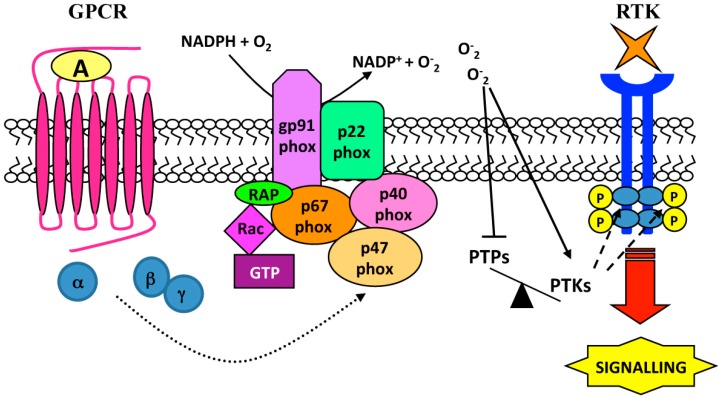
NADPH oxidase-dependent RTK transactivation. Agonist (A) stimulation of GPCRs induces p47^phox^ phosphorylation and NADPH oxidase activation, which generates reactive oxygen species (ROS) by O_2_ using NADPH as electron donor. ROS inactivate phosphotyrosine-phosphatases (PTPs) by oxidation of a cysteine in the catalytic domain, unbalancing intracellular phosphorylation equilibrium. The enhanced activity of phosphotyrosine-kinases (PTKs) mediates the trans-phosphorylation of tyrosines in the cytosolic region of RTK which, in turn, provide docking sites for assembly and activation of signaling complexes.

ROS are produced by a variety of extracellular stimuli such as growth factors, GPCR agonists, cytokines, ultraviolet radiation, increased osmolarity, and other cellular stresses [54]. Sources of their production include mitochondria [49], xanthine oxidase [55] and NADPH oxidase (Nox) family (Figure 2) [56], which generate superoxide anion (O_2_·), hydrogen peroxide (H_2_O_2_), and hydroxyl radical (OH·) involved in intracellular redox signaling. The pathophysiological effects of these molecules depend on their concentration, subcellular localization and the endogenous antioxidant status [57]. Plasma membrane-associated Nox family members are the only enzymes that generate ROS as their primary purpose in a highly regulated and spatial restricted manner, apparently suited for cell signaling [58,59] and are considered the main source of ROS acutely produced upon growth factor or cytokine stimulation [60,61]. Seven isoforms of the catalytic subunit (Nox1–5, Duox1 and 2) of NADPH oxidase, expressed in several cell types, have been identified.

In vascular smooth muscle cells (VSMCs) binding of Ang II to AT1 receptor triggers NADPH oxidase-dependent superoxide generation, which is responsible for EGFR phosphorylation at Y1068 [62]. In the same cells metalloprotease-dependent shedding of HB-EGF is required for H_2_O_2_-induced EGFR transactivation [63], and PAR1/PAR3 stimulation by thrombin induces Nox1-dependent generation of ROS, which results in EGFR transactivation via metalloprotease shedding of EGF-like ligands [64,65].

ROS generated by GPCR stimulation can result in transactivation of more RTKs, suggesting that instead of a single GPCR to single RTK cross-talk, a less selective more global RTK response to GPCR activation can occur. For instance, serotonin (5-HT) binds and activates six GPCRs and transactivates PDGFR-β receptor, as well as the tyrosine kinase receptor TrkB, through a NADPH oxidase-dependent mechanism, both in neuronal cultures and SH-SY5Y cells [66]. NADPH oxidase inhibitors prevent 5-HT-induced PDGFR-β phosphorylation, which is also dependent on PKC activity likely acting upstream of NADPH oxidase [66].

The mechanism of ROS-induced EGFR transactivation may involve non-receptor tyrosine kinases [13,67]. In VSMCs, Ang II induces ROS production via NADPH oxidase activation and EGFR transactivation [68]. In these cells c-Src acts as upstream regulator of EGFR transactivation and is also required for ROS generation [68]. Moreover, in human lung cancer cells Formyl-peptide receptor, a member of the GPCR family, stimulated with WKYMVm induces EGFR transactivation, which depends on c-Src activity. Apocynin, a NADPH oxidase inhibitor, or a siRNA against the catalytic subunit p22^phox^ of NADPH oxidase prevents EGFR transactivation and c-Src kinase activity [67]. In endothelial cells exposed to H_2_O_2_ the selective c-Src inhibitor PP2 prevents EGFR transactivation [69].

Several GPCR agonists activate Gq proteins family. The resulting GTP-Gαq complex activates PLCβ via interaction with the carboxy-terminal region of the enzyme [70]. The βγ subunits of G proteins can also stimulate PLCβ, as suggested by the observation that mitogenic GPCR ligands trigger the βγ-mediated stimulation of PLCβ2 and PLCβ3 [70]. Activate PLC isoforms catalyze the hydrolysis of phosphatydyl-inositol 4,5-bisphosphate (PIP2) to produce inositol 1,4,5-triphosphate (IP3) and diacylglycerol (DAG). IP3 triggers Ca^2+^ mobilization from endoplasmic reticulum stores leading to an increase of intracellular concentration of Ca^2+^ [71]. The most prominent intracellular targets of DAG are the classical and novel isoforms of PKC. In VSMCs ROS-induced EGFR transactivation may involve an increase of intracellular Ca^2+^ concentration, which is required for Ang II-dependent EGFR transactivation. Ang II induces ROS generation via NADPH oxidase activation [72] and since H_2_O_2_ has no effects on intracellular Ca^2+^, this ion may mediate ROS production which in turn triggers EGFR transactivation [73]. On the other hand, in mouse embryonic cells H_2_O_2_ increases intracellular Ca^2+^ concentration and the phosphorylation of PKC, ERKs, p38Mitogen-activated protein kinase (p38MAPK) and c-Jun *N*-terminal kinase (JNK), as well as EGFR transactivation stimulating, in turn, cell proliferation [74]. In WKYMVm-stimulated human fibroblasts, Ca^2+^-dependent PKCα isoform is required for the phosphorylation of the regulatory subunit p47^phox^ of NADPH oxidase [75].

Similar to EGFR, PDGFR can be activated not only by its cognate ligands but also by other stimuli in a ligand-independent manner. The transactivation of the PDGFRβ by GPCRs appears to involve ROS in VSMCs. In fact, in these cells hydrogen peroxide induces a ligand-independent tyrosine phosphorylation of PDGFR at Tyr1021, a PLCγ binding site, as well as the association of PKCδ with PDGFRβ and c-Src [76].

In human hepatocellular and pancreatic carcinoma cells the c-Met receptor becomes tyrosine phosphorylated in response to several GPCRs agonists, such as lysophosphatidic acid (LPA), bradichinin, thrombin, charbachol and endothelin. c-Met transactivation is prevented by reducing agents or treatment of cells with diphenylene iodonium [77], suggesting that it requires the acute production of ROS by membrane-bound NADPH oxidase. Phosphorylated tyrosines of c-Met provide docking sites for the triggering of intracellular signaling pathways [78]. In fact, WKYMVm, a Formyl-peptide receptor 2 agonist, induces the transactivation of c-Met in a human prostate epithelial cell line. The WKYMVm-induced phosphorylation of Y1313, Y1349 and Y1356 residues of c-Met in the intra-cytoplasmic domain elicits the activation of signal transducer and activator of transcription 3 (STAT3), PLC-γ1/PKCα and phosphatidylinositol-3kinase (PI3K)/Akt pathways, similarly to the molecular responses elicited by c-Met/HGF binding [79]. The critical role of NADPH oxidase-dependent ROS production in this crosstalk mechanism is supported by the finding that blockade of NADPH oxidase function prevents c-Met trans-phosphorylation and downstream signaling cascades [79].

FPR and the two RTKs, nerve growth factor (NGF) receptor neurotrophic tyrosine kinase receptor type 1 (TrkA) and EGFR, crosstalk each other to modulate pro-inflammatory mediators, to produce ROS, to activate MMP-9 and to up-regulate CD11b membrane integrin. In response to *N*-formyl-methionyl-leucyl-phenilalanine (*N*-fMLP), an FPR agonist, TrkA phosphorylation is prevented by EGFR inhibitors while EGFR phosphorylation is prevented by a TrkA inhibitor. Receptor crosstalk is Src- and ERK-dependent [80] (Table 2).

**Table 2:** RTKs transactivation: role of ROS.

Cell Lines	Stimulus	GPCR	Source of ROS	RTKs	Biological Responses	Ref.
VSMCs	Ang II	AT1	NADPH oxidase	EGFR	EGFR transactivation, ERKs activation, growth	[62]
SMC	Thrombin	PARs	NOX1	EGFR	EGFR transactivation, PI3K-Akt and ATF-1 activation, migration and proliferation, *N*-cadherin shedding mediated by MMP-9, ERKs activation	[64,65]
SH-SY5Y	5-HT	5-HTR	NADPH oxidase	PDGFR-β, TrkB	PDGFR-β transactivation, TrkB transactivation	[66]
Calu-6	WKYMVm	FPR2	NADPH oxidase	EGFR	EGFR transactivation, cell growth, STAT3 activation, PI3K/Akt activation	[67]
VSMCs	Ang II	AT1	NADPH oxidase	EGFR	EGFR transactivation, increase of intracellular Ca^2+^ concentration, MAPK activation	[72]
DAN-G, HepG2, HuH7	LPA, Bradykinin, Thrombin, Carbachol, Endothelin	LPA1, BDKRB1–2, PARs, mAChRs, EDNRs	NADPH oxidase	EGFR, c-Met	EGFR and c-Met transactivation, β-catenin nuclear traslocation, cell motility	[77]
PNT1A	WKYMVm	FPR2	NADPH oxidase	c-Met	c-Met transactivation, cell proliferation, STAT3 activation, PI3K/Akt activation, PLCγ/PKCα activation	[79]
Monocytes	*N*-fMLP	FPR	NADPH oxidase	EGFR, TrkA	EGFR and TrkA transactivation, CD11b membrane up-regulation	[80]

5-HT, Serotonin; 5-HTR, Serotonin receptor; Ang II, Angiotensin II; AT1, Angiotensin II receptor type 1; BDKRB1-2, Bradykinin receptor B1-2; c-Met, Hepatocyte growth factor receptor; Calu-6, Human lung cancer cells; DAN-G, Human pancreatic carcinoma cells; EDNRs, Endothelin receptors; EGFR, Epidermal growth factor receptor; FPR, *N*-formyl peptide receptor; FPR2, *N*-formyl peptide receptor 2; HepG2, Human hepatocyte cell line; HuH7, Human hepatocarcinoma cell line; LPA, Lysophosphatidic acid; LPA1, Lysophosphatidic acid receptor type 1; mAChRs, Muscarinic acetylcholine receptors; PARs, Protease activated receptors; PDGFR-β, Platelet-derived growth factor receptor; PNT1A, Human prostatic cell line; SH-SY5Y, Human neuronal cells; SMC, Human smooth muscle cells; TrkA, Neurotrophic tyrosine kinase receptor type 1; TrkB, Neurotrophic tyrosine kinase receptor type 2; VSMCs, Human vascular smooth muscle cells.

## 4. Ligand-Independent Mechanisms: Role of Intracellular Tyrosine Kinases

The absence of detectable amounts of EGF-like ligands after GPCR stimulation suggests that GPCR-induced EGFR transactivation can be also triggered by intracellular signaling pathways, which require the presence of Ca^2+^ ions as well as PKC and intracellular tyrosine kinases activation [9].

**Figure 3:**
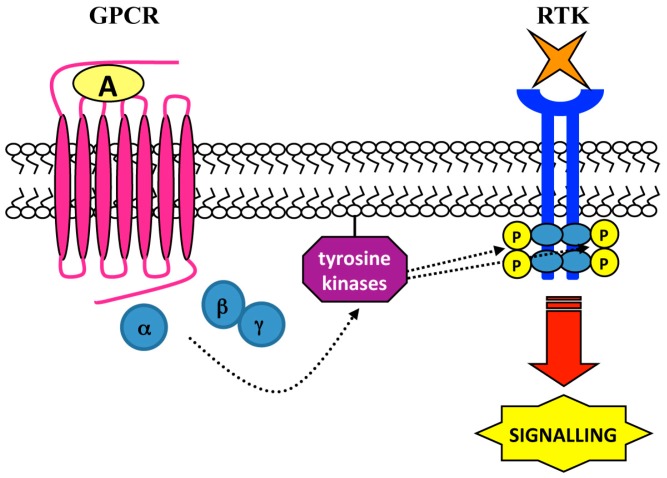
Tyrosine kinases-dependent RTK transactivation. RTK transactivation may be mediated by non-receptor protein tyrosine kinases. GPCR-activated members of c-Src family induce a ligand-independent transactivation of a RTK via trans-phosphorylation of cytosolic tyrosines, which provide docking sites for triggering intracellular signaling cascades.

Src family tyrosine kinases are an integral component of the signal transduction apparatus employed by RTKs, playing a key role in cellular growth and malignant transformation. Several molecular mechanisms allow GPCRs to activate Src family kinases, and conversely Src activity plays a central role in controlling GPCR responses. In many cases Src family kinases are associated with GPCRs through direct interaction with cytoplasmic receptor domains, which contain consensus SH3 domain-binding motifs or proline-rich motifs within their third intracellular loops or *C*-terminal tails, or by binding to GPCR-associated proteins, such as heterotrimeric G-protein subunits or β-arrestins. Src family kinases are also activated by GPCRs [81].

c-Src is regulated by Gβγ subunits and can interact with β-arrestin, which binds a number of signaling proteins. In some cell types the formation of β-arrestin-Src complex can requires GPCR phosphorylation. In fact, in human epidermoid carcinoma cells isoproterenol triggers tyrosine phosphorylation of β2-adrenergic receptor (β2AR) playing a role in β2AR-Src interaction [82]. In PC3 cells binding of neurotensin to Neurotensin receptors (NTRs), which are members of GPCR family, induces c-Src-dependent EGFR phosphorylation at Y845 [83], and several GPCR agonists induce rapid and transient activation of Src-family members in a variety of cell types (Figure 3) [84]. Stimulation of α2A-adrenergic receptor (AR) results in EGFR transactivation and ERKs activation which requires c-Src activity and, in turn, the induction of HB-EGF shedding mediated by metalloproteases [85]. Alternatively, in response to GPCR-induced ROS generation, Src-family members directly phosphorylate EGFR [85].

In C9 cells stimulation of AT1 with Ang II results in the association of Pyk2 with Src and EGFR, as well as in ERKs activation. Src, Pyk2 and Ang II-induced ERKs phosphorylation mediated EGFR transactivation actin upstream to EGFR [86]. Also stimulation of the V2 vasopressin receptor (V2R) leads to the ERKs activation, through a c-Src-dependent, metalloprotease-mediated shedding of a factor activating Insulin Growth Factor Receptor (IGFR), which requires the presence of β-arrestins [87]. c-Src acts upstream of the metalloprotease activation and is required for the release of the IGFR-activating factor, whereas β-arrestins act downstream of the IGFR transactivation. The engagement of β-arrestins by IGFR, but not by V2R, is necessary to promote the vasopressin-stimulated ERKs activation. This role of β-arrestins is not limited to the V2R but also to other unrelated GPCRs, indicating that it may be a general mechanism shared among GPCRs [87].

Formyl-peptide receptors 1, 2 and 3 (FPR1, 2 and 3) form a subgroup of receptors linked to inhibitory G-proteins [88]. Their activation by specific ligands triggers distinct signaling cascades in several cell types [89,90]. Stimulation of CaLu6 cells with the FPR2 agonist WKYMVm induces EGFR transactivation, p47^phox^ phosphorylation, NADPH-oxidase-dependent superoxide generation and c-Src kinase activity. Phosphorylated tyrosine residues of EGFR recruit and trigger the STAT3 pathway. The role of c-Src and ROS in this crosstalk mechanism is corroborated by the observation that a c-Src inhibitor or the blockade of NADPH oxidase function hampers EGFR transactivation. Furthermore, the NADPH oxidase inhibitor apocynin or a siRNA against the catalytic subunit p22^phox^, prevents WKYMVm-induced c-Src activation [67].

Fyn, a member of the Src family kinase, plays a key role in regulating signaling events between Adenosine receptor and Trk. Fyn and Trk are colocalized in a juxtanuclear membrane compartment and Fyn expression is sufficient to allow transactivation of Trk by adenosine. These observations indicate that Fyn is activated by Adenosine receptor stimulation and is responsible for transactivation of Trk receptors on intracellular membranes [91]. c-Src activity is also required in serotonin-induced PDGFRβ phosphorylation through 5-hydroxytryptamine 1A (5-HT1A) receptors in SH-SY5Y neuroblastoma cells and in primary cortical neurons. The receptor transactivation is pertussis toxin (PTX)-sensitive and Src-dependent and requires PLC activity, intracellular calcium signaling and NADPH oxidase activation [92].

In many cases RTKs and GPCRs form a complex on which signals can be integrated to produce more efficient stimulation of intracellular pathways. PDGF and sphingosine 1-phosphate (S1P) induce the recruitment of c-Src to the PDGFRβ-sphingosine 1-phosphate receptor 1 (S1P1) complex. This drives to the tyrosine phosphorylation of Grb2-associated-binding protein 1 (Gab1), which depends on G protein and c-Src activation, as well as the accumulation of dynamin II at the membrane, which is required for endocytosis of the PDGFRβ-GPCR complex [93]. In other observations, ROS and Src-family kinases seems to act downstream of PDGFR to enhance PDGF-mediated tyrosine phosphorylation of various signaling intermediates [94]. The formation of functional complexes between RTKs and GPCRs is observed in different cell types [95,96]. For instance, in airway smooth muscle cells PDGFRβ and S1P1 form an active complex. The respective ligands (PDGF or S1P) do not induce changes in the association of these receptors in the complex and S1P does not induce tyrosine phosphorylation of PDGFRβ [95]. However, over-expression of S1P1 enhances the PDGF-stimulated activation of the ERKs pathway. [10]. In COS-7 cells, β2-adrenergic receptor (β2AR) stimulation induces EGFR dimerization, tyrosine autophosphorylation and EGFR internalization. Isoproterenol exposure promotes the formation of a multireceptor complex containing β2AR and the transactivated EGFR. Selective inhibitors of Src kinases prevent β2AR-mediated EGFR phosphorylation, indicating that this kinase is required for EGFR transactivation [97]. Similarly, in gastric mucosal cells stimulated with isoproterenol, PP2 prevents EGFR transactivation and gastric mucin secretion [98]. Src family kinases are also required in the insulin-like growth factor (IGF)-mediated transactivation of active pituitary adenylate cyclase-activating peptide type 1 receptor (PAC1R), which is constitutively associated to the insulin-like growth factor 1 receptor (IGF-1R) and is involved in IGF-1-induced survival of neurons [99].

Other functional complexes between GPCRs and RTKs do not require the action of intracellular tyrosine kinases for the transactivation mechanism. This is the case of TrkA receptor/neurotrophic tyrosine kinase receptor type 1 (TrkA/NTRK1) association [100] and of S1P1/VEGFR2, which forms a complex associated to PKC and ERKs [101].

Much less is known regarding the ability of ROS and Src-family members to indirectly and chronically activate monomeric PDGFRα. However, the possible involvement of metalloproteases in the PDGFR transactivation mechanism and, in turn, the applicability of the TMPS model is not clear. The ligand-dependent mechanism requires the existence of latent growth factors that could be activated by proteolytic cleavage. Although latent ligands of PDGFR (platelet-derived growth factor C and platelet-derived growth factor D) have been discovered, their possible partecipation in the PDGFR transactivation has not yet been investigated [102].

Activation of intracellular tyrosine kinases can require the presence of Ca^2+^ ions. In cardiomyocytes the Endothelin-1 (ET)-mediated activation of the tyrosine kinase Pyk2 is abrogated by chelating Ca^2+^ or by down-regulation of PKC, whereas ET-1-mediated transactivation of EGFR is solely dependent on PKC, suggesting the existence of two distinct tyrosine kinase pathways requiring Pyk2 or PKC activation downstream from GPCR [103] (Table 3).

**Table 3:** RTKs transactivation: role of tyrosine kinases.

Cell Lines	Stimulus	GPCR	Tyrosine Kinases	RTK	Biological Responses	Ref.
PC3	Neurotensin	NTRs	c-Src	EGFR	EGFR transactivation, cell proliferation, DNA synthesis, STAT5-b activation	[83]
COS-7	α2-AR agonists	α2-AR	c-Src	EGFR	EGFR transactivation, ERK activation	[85]
C9	Ang II	AT1	c-Src/Pyk2	EGFR	EGFR transactivation, ERKs phosphorilation	[86]
HEK293	AVP	V2R	c-Src	IGFR	IGFR transactivation, ERKs activation	[87]
PC12-615	Adenosine, GCS21680	Adenosine receptor	Fyn	TrkA	TrkA transactivation.	[91]
SH-SY5Y	5-HT	5-HT1A	c-Src	PDGFR-β	PDGFR-β transactivation	[92]
COS-7	Isoproterenol	β2AR	c-Src	EGFR	EGFR transactivation, ERKs activation	[97]
Gastric mucosal cells	Isoproterenol	β2-AR	c-Src	EGFR	EGFR transactivation and regulation of gastric mucin secretion	[98]
Cardiomyocytes	Endothelin-1	ET-1	Pyk2	EGFR	EGFR transactivation, MAPK activation	[103]

5-HT, Serotonin; 5-HT1A, Serotonin receptor; Ang II, Angiotensin II; AT1, Angiotensin II receptor type 1; AVP, Vasopressin; C9, Rat hepatic cells; COS-7, Fibroblast-like cell line; EGFR, Epidermal growth factor receptor; ET-1, Endothelin receptor; HEK293, Human embryonic kidney 293 cells; IGFR, Insulin-like growth factor 1 receptor; NTSR, Neurotensin receptor; PC12-615, Rat pheochromocytoma cell line; PC3, Human prostate carcinoma cells; PDGFR-β, Platelet-derived growth factor receptor; SH-SY5Y, Human neuronal cells; TrkA, Neutrophic tyrosine kinase receptor type 1; V2R, Vasopressin receptor; VSMCs, Vascular smooth muscle cells; β2AR, β2-Adrenergic receptors.

## 5. Role of G Proteins and β-Arrestins in Transactivation

RTKs can use G proteins to induce activation of signaling pathways and this mechanism is distinct from RTK transactivation, in which the G protein functions upstream of the RTK to transmit signals to effectors. Further studies reveal that Gi is constitutively associated with IGF-1R and that stimulation of cells with insulin results in a reduction in the amount of Gβγ associated with IGF-1R [10]. On the other hand IGF-1 binds to the Gi coupled chemokine receptor type 4 (CXCR4) in the absence of Chemokine 12 (CXCL12), the natural ligand of CXCR4, to promote migration of breast cancer cells [104]. This mechanism involves the regulation of Gαi and βγ subunits by IGF-1 [104].

Other RTK-G protein partnerships include PDGFRβ signaling in HEK293 cells over-expressing PDGFRβ, where PDGF stimulates the tyrosine phosphorylation of Gαi [10]. The interaction between RTKs and G protein is not restricted to Gi, thereby demonstrating wider significance of this type of regulation. In fact, EGF stimulates the tyrosine phosphorylation of Gαs, which is more effective at stimulating adenylyl cyclase than its non-phosphorylated counterpart [10]. Moreover, Gα13 is essential for EGFR- and PDGFR-induced migration of fibroblasts and endothelial cells in response to EGF and PDGF, respectively [105]. This suggests that Gα13 is a critical signal transducer for RTKs, as well as for GPCRs.

There is no evidence that RTKs can directly activate G-proteins in a manner similar to a GPCR. PTX prevents Gi-proteins functional coupling with the GPCR by reducing activation of Gi induced by GPCR agonists. RTKs might use activated Gi provided by a constitutively active GPCR in a platform complex with the RTK and in this model, PTX interrupts the signaling from the RTK–GPCR association by preventing activation of Gi-protein by the GPCR [10].

β-Arrestins participate in cellular responses to growth factors, denoting additional evidence for a role of GPCRs in regulating RTK signal transmission. They are implicated in GPCRs desensitization but are also clathrin adaptor proteins, which can function as regulators of GPCR-dependent signaling. β-Arrestins can associate with the PDGFRβ, TrkA, IGF-1R and EGFR in either a ligand-dependent or -independent manner [106,107,108], thereby suggesting either a unique interaction between RTK and β-arrestin or a potential role for β-arrestin associated with a GPCR in a complex with RTK [10].

β-Arrestins are involved in the GPCR-mediated transactivation of several RTKs, such as EGFR. For instance, β1AR specific agonists trigger β-arrestin-dependent EGFR phosphorylation, which is independent by G protein activation and requires GRK5/6 activity [109]. β1AR and EGFR form a complex at the plasma membrane and their interaction depends on β1AR phosphorylation by GRK and on recruitment of β-arrestin, which is required to maintain prolonged β1AR-EGFR interaction and to retain ERKs activation in the cytosol [18]. A genetic variant of α1AR triggers a β-arrestin1/c-Src/MMP/EGFR/ERK-dependent hyperproliferation of cardiomyoblasts, which is constitutive, and Gq independent, as well as a Gq/EGFR/STAT-dependent hypertrophy, which is induced by α1AR agonists. These two distinct EGFR transactivation-dependent cascades induce the transition of cardiomyoblasts to fibroblast-like cells [23]. Urotensin II stimulation in an animal model of transverse aortic construction [14] and GPR54 stimulation by Kisspeptin-10 in human invasive breast carcinoma cells [110] represent other examples of β-arrestin-dependent EGFR transactivation.

β-Arrestins are also recruited to the IGF-1R, mediating intracellular signaling cascades. GRK plays a key role in this molecular mechanism, as demonstrated by the observation that silencing GRK expression abolished IGF-mediated ERKs phosphorylation and Akt activation [111]. Chronic insulin treatment induces to enhance β-arrestin1 degradation, which is associated with a decreased IGF-1, LPA and MAPK signaling, suggesting that insulin treatment can impair GPCR signaling [107]. A brief stimulation of µ opioid receptor (MOPR), a member of the GPCR family, with its specific agonist transactivates IGF-1R in SH-SY5Y cells. Small interfering siRNA for β-arrestin2 uncouple the cross-talk between the two receptors [112]. On the other hand, MMP-dependent IGF-1R transactivation induced by V2R stimulation requires c-Src activity, which acts upstream of the MMP activation, as well as β-arrestins, which act downstream of the IGF-1R transactivation [87]. Moreover, S1P induces recruitment of β-arrestin to S1P1/S1P3 receptor, c-Src activation and the binding of S1P1/S1P3 with Flk-1, increasing Flk-1 phosphorylation and, in turn, embryonic stem cell proliferation [113].

## 6. GPCR-Mediated Transactivation of Serine/Threonine kinase (S/TK) Receptors and Toll-Like Receptors (TLRs)

**Figure 4:**
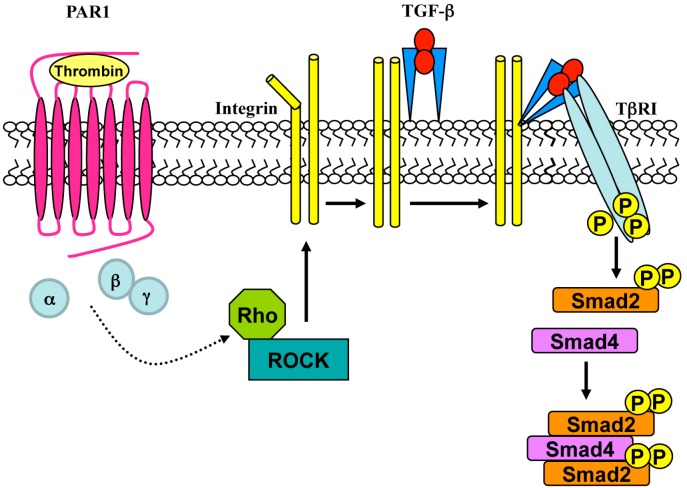
GPCR-mediated TβRI transactivation. Stimulation of protease activated receptor 1 (PAR1) with thrombin induces the activation of Rho/ROCK which, in turn, triggers integrin-mediated TGF-β activation. The activated ligand binds to TβRI promoting the phosphorylation of Smad2 and the formation of pSmad activated complexes.

GPCR signaling involving receptor transactivation has been limited to RTKs but it can be extended to S/TK receptors, such as TGF-β receptor (TβRI). Several GPCR agonists triggering TβRI transactivation have been identified. In VSMCs Endothelin-1 and thrombin bind to their specific GPCRs inducing a temporal increase of *C*-terminal phosphorylated Smad transcription factor (pSmad2) [114,115], which is the immediate downstream product of activation of the S/TK activity of TβRI (Figure 4). The thrombin-mediated increase of pSmad2 is insensitive to cycloheximide, suggesting a transactivation mechanism independent of gene activation and de novo protein synthesis [1]. In human epithelial cells, LPA induces αVβ6-integrin-mediated TGF-β activation, via both Ras homolog member A (RhoA) and Ras homolog kinase (ROCK), which is elicited by Gαq associated to LPA2 receptor, a member of the GPCR family [116]. Similarly, in mice epithelial cells thrombin and other ligands of PAR1 activate TGF-β in a αVβ6-integrin-dependent manner via RhoA and ROCK [117]. Stimulation with either LPA or thrombin induces a time-dependent generation of pSmad2, which is completely prevented by an αVβ6-integrin-specific neutralizing antibody [116,117]. In VSMCs, PAR1-mediated transactivation of TβRI involves cytoskeletal rearrangement, ROCK and cell-surface Arg–Gly–Asp (RGD)-binding integrins. The resulting increase of pSmad2 phosphorylation plays a key role in proteoglycan synthesis [118]. In mouse VSMCs binding of Ang II to AT1 receptor induces TβRI transactivation and small mother against decapentaplegic 2C (Smad2C) phosphorylation, as well as the induction of collagen type I expression. The Ang II-iduced pSmad2 generation is abolished by αVβ3-integrin-specific neutralizing antibody, indicating that S/TK receptors transactivation requires cell-type specific isoforms of integrins [119].

Another study provides evidence for S/TK Bone Morphogenetic Protein Receptor 1A (BMPR1A) transactivation by the GPCR agonist 5-HT. In bovine and human pulmonary artery smooth muscle cells, serotonin activates Smad 1/5/8 through the 5-HT 1B/1D receptor, a member of the GPCR family, and induces their translocation from cytoplasm to the nucleus. Smads phosphorylation depends on RhoA and ROCK signaling and is prevented by blockade of 5-HT 1B/1D receptor and by the expression of a negative dominant BMPR1A, suggesting that it requires the activation of both receptors [120].

There is also evidence for a crosstalk between GPCR and TLR signaling pathways. Mammalian TLRs recognize pathogen-associated molecular patterns and trigger intracellular signaling cascades in dendritic cells (DC). These include modified chemokine and cytokine production, altered chemokine receptor expression, and changes in signaling through GPCRs by altering the expression of regulator of G protein signaling (RGS) proteins [121]. In macrophage cells LPA, bombesin, Ang I and II, and bradikinin bind their respective GPCRs inducing a rapid Neu1 activity, which is prevented by PTX and MMP inhibitors. In particular, the bombesin-related Neuromedin B Receptor (NMBR) forms a complex tethered to the glycosylated TLR4, supporting the crosstalk between GPCR and TLR signaling pathways [122]. Thymoquinone also activates Neu1 and Neu4 sialidases on the cell-surface of DCs and macrophages via GPCR and Gαi, and TLR 2/3/4 form a signaling platform with GPCR, Gαi, MMP-9 and Neu1 (or Neu4) sialidase [123].

TLRs dimerization is essential for the ligand-induced activation and is a prerequisite to facilitate myeloid differentiation primary response 88 (Myd88)/TLR complex formation and subsequent intracellular signaling. In murine macrophages, Neu-1 sialidase and MMP-9 crosstalk, in alliance with NMBR tethered to TLR7 and 9, forms a GPCR signaling platform, which plays a key role in ligand activation of TLRs. Ligand binding to TLR7 and 9 triggers conformational changes to potentiate GPCR signaling via MMP-9 activation and Gαi subunits to induce Neu1 sialidase, which hydrolizes syalil residues linked to β-galattosidase, triggering TLRs dimeric complex formation and MyD88/TLR recruitment [124].

On the other hand, in murine macrophages the endotoxin lipoplysaccaride (LPS) binds to TLR4 and potentiates GPCR signaling via Gαi proteins and MMP-9, inducing Neu1 sialidase activity. The Neu1-MMP-9 crosstalk in alliance with TLR4 and GPCR on cell surface generates a molecular signaling platform that is essential for ligand-induced TLR activation. The signaling paradigm suggests a conformational change of the receptor, as a result of ligand binding, to initiate GPCR signaling via Gαi proteins and MMP-9 activation to induce Neu1. Activated Neu1 is tethered to TLR4 and hydrolizes syalil residues at the ectodomain of the receptor [125].

## 7. RTK-Mediated GPCR Transactivation

Signal transduction cascades triggered by several RTKs are mediated, at least in part, by transactivation of GPCRs, suggesting a reciprocal transactivation between the two classes of receptors. Molecular models of GPCR transactivation by RTK ligands depend on the nature of the GPCR–RTK partners and are similar to those employed by GPCR agonists to transactivate RTKs. In some cases, GPCR transactivation occurs in a ligand-dependent manner through synthesis and secretion of a ligand of the transactivated GPCR. This ligand activates the GPCR in an autocrine and/or paracrine manner [126,127,128,129]. In other GPCR-RTK partnerships, the transactivation of GPCRs by RTK agonists occurs in a ligand-independent manner and requires the formation of GPCR-RTK complexes, which might also arise from their interaction with intracellular scaffolding proteins, as well as the phosphorylation of the transactivated GPCRs [130]. Enzymatic activation and/or transcriptional up-regulation are required for the synthesis and the extracellular accumulation of GPCR ligands, upon RTK stimulation. For instance, S1P1 is transactivated by NGF as a consequence of production of S1P [7]. Stimulation with NGF triggers both sphingosine kinase 1 (SphK1) plasma membrane translocation and SphK1 activation, which enhances synthesis and secretion of S1P and in turn, S1P1 activation [7].

**Figure 5:**
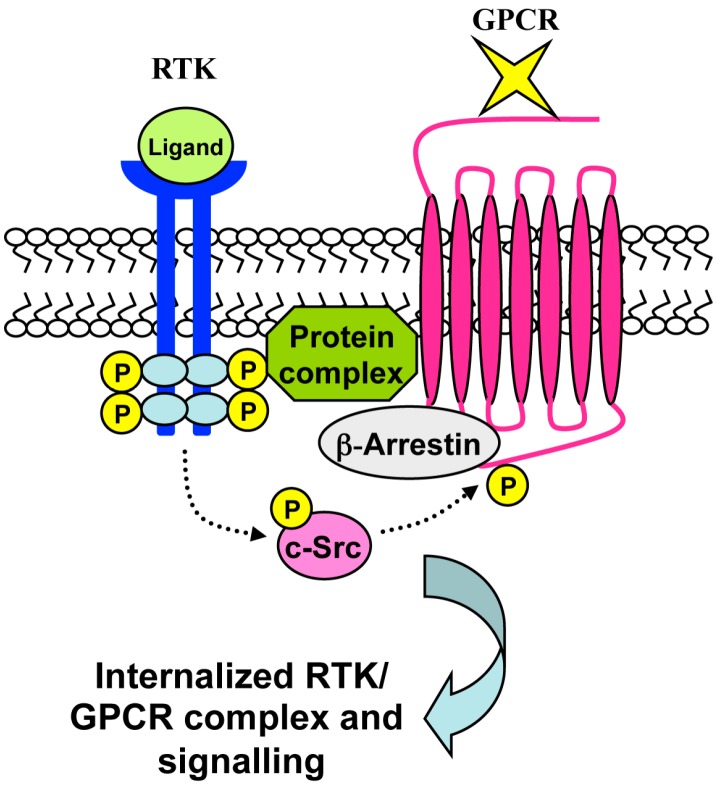
Ligand-independent RTK-mediated GPCR transactivation. The transactivation of GPCRs by RTK agonists can occur in a ligand-independent manner and requires the formation of GPCR–RTK complexes. RTK stimulation triggers GPCR transactivation through the generation of a molecular protein complex. Several proteins contribute to this event, such as c-Src, which promotes the phosphorylation of cytoplasmic tyrosines residues of GPCRs. This event is crucial for β-arrestin recruitment, which, in turns, promotes the internalization of the RTK/GPCR complex and intracellular signaling cascades.

GPCR phosphorylation can represent a mechanism that contribute to cellular effects induced by RTK agonists. For instance, insulin promotes phosphorylation of β2-adrenoceptors on a tyrosine residue, which does not transactivate β2-adrenoceptors but results in an increase in receptor functional activity [131]. The modulation of β2-adrenergic receptors function by insulin requires the coordinate action of at least three pathways [7] which involve (i) β2-adrenoceptors phosphorylation on tyrosine residues mediated by the insulin receptor [132]; (ii) the activation of Akt/PKB pathway, which phosphorylates β2-adrenergic receptors on serine residues [133] and (iii) the activation of c-Src by insulin, which leads to a further stimulation of phosphatidylinositol-3kinase (PI3K) [134]. IGF-I induces tyrosine phosphorylation of β2-adrenoceptors at different sites than insulin [135]. Similarly, α1B-adrenoceptor phosphorylation can be triggered by EGFR and PDGFR activation [136] and the actions of EGF and PDGF are prevented by PTX [137], indicating that the inhibition of G protein function alters RTK actions. The general mechanism contemplates an integrated signaling unit composed by RTKs and GPCRs, capable of responding to RTK agonists utilizing GPCRs as accessory elements and employing both, G proteins and the tyrosine phosphorylation pathways. This integrated signaling [98,138] can involve recruiting of intracellular protein kinases (c-Src or PI3K) or adaptor proteins (β-arrestins, Grb2 or Gab1), which could facilitate the combined actions of RTKs and GPCRs (Figure 5).

## 8. Conclusions

GPCR agonists act as efficient cellular growth factors in several cell types, activating a highly interconnected signaling network. Activated GPCRs interact with one or more G proteins, or trigger intracellular cascades through G protein-independent mechanisms. Furthermore, hetrotrimeric G proteins regulate monomeric G proteins, suggesting that heptahelical receptors act as part of multi-protein signaling complexes. RTKs form complexes with GPCRs, which can supply G-protein for use by RTKs and, in other cases, distinct RTKs can be directly associated with heterotrimeric G-proteins. Combinations of RTK inhibitors and GPCR-specific ligands that prevent Gi function can represent the most efficient method to inhibit signaling from RTK-GPCR platform complexes.

GPCRs transduce signals through both the synthesis of second messengers and the recruitment of non-receptor tyrosine kinases and/or RTKs on plasma membrane. GPCR-mediated RTK transactivation can occur by different molecular mechanisms which include ADAM- or MMP-dependent release of precursor forms of RTK agonists, activation of membrane associated non-receptor tyrosine kinases (Src, Lyn, Pyk, Fyn), and ROS generation, mainly through the activation of the NADPH oxidase complex. Nox proteins are the only enzymes that generate ROS in a highly regulated manner, suited to modulate intracellular cellular communication. Nox2, a member of the NADPH oxidase enzyme family, is activated by several GPCR agonists, by cytokines and by growth factors. The molecular mechanism for RTK transactivation can involve inactivation of PTPs by ROS which, in turn, induces an increase of protein tyrosine kinase activity. Targeting ROS-sensitive mechanisms with selective drugs and the further characterization of the molecular mechanisms involved in ROS-mediated signal transduction via RTK activation can provide new targets for adequate therapies.

Many of the pathways triggered by GPCR agonists are also engaged by RTK ligands, providing broad changes of crosstalk between heterologous receptors. The crosstalk can occur at proximal level (receptor to receptor) and a high degree of selectivity and specificity is required for GPCR-mediated transactivation of a distinct RTK, or downstream of intracellular signaling pathways involving members of Src family tyrosine kinases, ERKs, p38MAPK, JNK and other kinases. The crosstalk can also occur between receptors belonging to the same family, as the case of the activation of IGF-1R which can induce EGFR transactivation and MAPK activation.

Autophosphorylated RTKs provide docking sites for the recruitment of signaling proteins via SH2 and PTB sites. Similarly, RTK-mediated phosphorylation of GPCRs induces the formation of SH2 sites on heptahelical receptors, providing the notion that RTKs can supply similar scaffold properties to GPCRs. These receptor-based signaling complexes differ among different cell types. GPCR transactivation by RTK agonists has been proved only for a few GPCR-RTK partnerships and needs to be generalized. In several human diseases over-expression of many RTKs, GPCRs and/or their enhanced activity, as well as increased levels of growth factors has been observed, which have been associated with increased cell proliferation and migration. Various pharmacological approaches have been developed to target RTKs in different human cancers. The dissection of the molecular mechanisms responsible of GPCR transactivation by RTK ligands might lead to the development of new drugs able to block RTK signaling and RTK-dependent GPCR transactivation, by antagonists or inverse agonists of the corresponding GPCRs.

GPCRs can also transactivate S/TK cell surface receptors, such as TβRI which efficiently binds TGF-β. This observation suggests a possible shared mechanism of transactivation in which a GPCR inhibitor can prevent both tyrosine kinase and S/TK receptor transactivation. It is not clear if GPCR activation by multiple GPCR agonists transactivates many RTKs and S/TK receptors through the activation of multiple signaling cascades. The identification of specific molecular targets in these pathways, linked to specific human diseases, can provide new approaches for therapeutic intervention.

Crosstalk between GPCRs and RTKs provides wider changes for the identification of new drugs for the treatment of human diseases attributed to an increased level of growth factors or to modulation of RTKs activity, challenging current thinking in the definition of pharmacological targets.
